# Supplementary material for: Biogenesis of phased siRNAs on membrane-bound polysomes in Arabidopsis
Source: eLife. 2016 Dec 12;5:e22750. doi: 10.7554/eLife.22750 (PMC5207768; doi:10.7554/eLife.22750)
Supplement: Supplementary file 5. — DOI: http://dx.doi.org/10.7554/eLife.22750.019 [file elife-22750-supp5.docx]

| **name** | **sequence** | **purpose** |
| --- | --- | --- |
| rev-raceR | GCTGCCTTCCTAATCCATACACTACTTCACCATT | 5’ RACE RT PCR |
| phb-raceR | GACACTCCAGGCATCCAGATCAACATGA | 5’ RACE RT PCR |
| cna-raceR | GCCGCCATTGTTGTCTTCTGTGCAA | 5’ RACE RT PCR |
| csd2-raceR | GTCAAGCCAATCACACCACATGCCAA | 5’ RACE RT PCR |
| \| cuc1 raceR \| CAGAGAGTAAACGGCCACACACTCAC \| \| --- \| --- \| | CAGAGAGTAAACGGCCACACACTCAC | 5’ RACE RT PCR |
| Oligo dT linkerM | /5Phos/GAT CGT CGG ACT GTA GAA CTC TGA ACC TGT CGG TGG TCG CCG TAT CAT T/iSp18/CA CTC A/iSp18/CC TTG GCA CCC GAG AAT TCC ATT TTT TTT TTT TTT TTT TTT VN | Ribo-seq |
| rDNA dep-1 | /5BioTEG/TGGCCGAGGGCACGTCTGCCTGGGTGTC | Ribo-seq; depletion of rRNA |
| rDNA dep-2 | /5BioTEG/TGGTCGGCTTGTCCCTTCGGTCGGC | Ribo-seq; depletion of rRNA |
| rDNA dep-3 | /5BioTEG/GCGGGTGCACCGCCGACCGACCTTG | Ribo-seq; depletion of rRNA |
| rDNA dep-4 | /5BioTEG/TCTGATGATTCATGATAACTCGACGGATCGC | Ribo-seq; depletion of rRNA |
| rDNA dep-5 | /5BioTEG/GCCAAGGATGTTTTCATTAATCAAGAAC | Ribo-seq; depletion of rRNA |
| rDNA dep-6 | /5BioTEG/AACCCCTGTTTTTGGTCCCAAGGCTCGC | Ribo-seq; depletion of rRNA |
| rDNA dep-7 | /5BioTEG/TGCCGGCCGGGGGACGGACTGGG | Ribo-seq; depletion of rRNA |
| rDNA dep-8 | /5BioTEG/ATTCAGCCCTTTGTCGCTAAGATTCG | Ribo-seq; depletion of rRNA |
| rDNA dep-9 | /5BioTEG/GCCGCTCACGCCCGGTCGTACTC | Ribo-seq; depletion of rRNA |
| rDNA dep-10 | /5BioTEG/CAATGATTAGAGGCATTGGGGGCGC | Ribo-seq; depletion of rRNA |
| miR167AS | TAGATCATGTTGGCAGTTTCA | miRNA northern blot |
| miR156AS | GTGCTCTCTTTCTTCTGTCA | miRNA northern blot |
| miR398AS | AAGGGGTGACCTGAGAACACA | miRNA northern blot |
| miR169AS | CGGCAAGTCATCCTTGGCTCA | miRNA northern blot |
| U6 | AGGGGCCATGCTAATCTTCTCTG | miRNA northern blot |
| PHB-T7-F | GGCACGTAATACGACTCACTATAGGGGTCTGTGGTCGTGAGTGGTC | In vitro transcription |
| PHB-R | GCCAAGATGGACGATCTTTG | In vitro transcription |

Supplementary File 5. Oligonucleotides used in this study
